# Development and validation of the ACE tool: assessing medical trainees’ competency in evidence based medicine

**DOI:** 10.1186/1472-6920-14-114

**Published:** 2014-06-09

**Authors:** Dragan Ilic, Rusli Bin Nordin, Paul Glasziou, Julie K Tilson, Elmer Villanueva

**Affiliations:** 1 Department of Epidemiology & Preventive Medicine, School of Public Health & Preventive Medicine, Monash University, Level 6, The Alfred Centre, 99 Commercial Rd, Melbourne VIC 3004, Australia; 2 Jeffrey Cheah School of Medicine and Health Sciences, Monash University, Johor Bahru, Malaysia; 3 Faculty of Health Sciences and Medicine, Bond University, Robina, Australia; 4 Division of Biokinesiology and Physical Therapy, University of Southern California, Los Angeles, USA; 5 Gippsland Medical School, Monash University, Churchill, Australia

**Keywords:** Evidence based medicine, Assessment, Medical students

## Abstract

**Background:**

While a variety of instruments have been developed to assess knowledge and skills in evidence based medicine (EBM), few assess all aspects of EBM - including knowledge, skills attitudes and behaviour - or have been psychometrically evaluated. The aim of this study was to develop and validate an instrument that evaluates medical trainees’ competency in EBM across knowledge, skills and attitude.

**Methods:**

The ‘Assessing Competency in EBM’ (ACE) tool was developed by the authors, with content and face validity assessed by expert opinion. A cross-sectional sample of 342 medical trainees representing ‘novice’, ‘intermediate’ and ‘advanced’ EBM trainees were recruited to complete the ACE tool. Construct validity, item difficulty, internal reliability and item discrimination were analysed.

**Results:**

We recruited 98 EBM-novice, 108 EBM-intermediate and 136 EBM-advanced participants. A statistically significant difference in the total ACE score was observed and corresponded to the level of training: on a 0-15-point test, the mean ACE scores were 8.6 for EBM-novice; 9.5 for EBM-intermediate; and 10.4 for EBM-advanced (p < 0.0001). Individual item discrimination was excellent (Item Discrimination Index ranging from 0.37 to 0.84), with internal reliability consistent across all but three items (Item Total Correlations were all positive ranging from 0.14 to 0.20).

**Conclusion:**

The 15-item ACE tool is a reliable and valid instrument to assess medical trainees’ competency in EBM. The ACE tool provides a novel assessment that measures user performance across the four main steps of EBM. To provide a complete suite of instruments to assess EBM competency across various patient scenarios, future refinement of the ACE instrument should include further scenarios across harm, diagnosis and prognosis.

## Background

Evidence based medicine (EBM) is now well established as a discipline across a variety of medical, allied and health sciences curricula. EBM provides users with the ability to integrate evidence into decision making alongside clinical expertise and patient values [1]. EBM integrates knowledge and skills from a variety of sub-disciplines including clinical epidemiology, information literacy, knowledge management and biostatistics. Practicing ‘EBM’ requires that users are skilled across a 5-step process that includes; (i) construction of an answerable question from the clinical scenario, (ii) systematic retrieval of the best available evidence, (iii) critical appraisal of the evidence for validity, clinical relevance and applicability, (iv) application of results, and (v) evaluation of performance [2].

In 2011 the Sicily statement on classification and development of evidence-based practice learning assessment tools was developed [3]. The classification rubric for EBP assessment tools in education (CREATE) provides guidance when developing new EBM-related assessments by classifying assessment categories (reaction to educational experience, attitudes, self-efficacy, knowledge, skills, behaviours and benefit to patients) and types (self-report, cognitive testing, performance assessment, activity monitoring and patient orientated outcomes) with the five EBM steps [3]. The CREATE framework suggests that reaction to educational experience, self-efficacy and attitudes towards EBM are best assessed via self-report. Conversely, active monitoring and patient-orientated outcomes are believed to best assess EBM behaviour and benefit to patients [3]. EBM related knowledge and skills are the only assessment categories that are best examined via performance, or cognitive, assessment [3].

A variety of instruments have been developed to assess competency in (EBM). A 2006 systematic review identified 104 unique instruments, of which validity was established for only 53% [4]. Few instruments have been developed to assess all aspects of EBM, including knowledge, skills attitudes and behaviour, or been psychometrically evaluated. The Fresno test [5] and the Berlin Questionnaire [6] represent the only two instruments developed to date that assess knowledge and skills across 3 of the 5 EBM steps (ask, acquire and appraise) [3].

The Fresno test was developed and validated to assess medical professionals’ EBM skills and knowledge [5]. It provides users with a choice of two clinical scenarios, of which users are required to choose one in order to answer 12 open-ended questions. The test takes approximately 60 minutes to complete, and reports high inter-rater reliability regarding assessment of items. The original Fresno test has been adapted for use in other health disciplines including physical and occupational therapy with acceptable reliability and validity [7,8]. The Berlin Questionnaire is a 15-item multiple choice assessment also developed and validated to assess medical professionals’ EBM skills and knowledge [6]. The Berlin Questionnaire was designed to measure deep learning, with the emphasis on the application of existing EBM knowledge and skills [6]. Deep learning in this context assesses a user’s ability to actively search for understanding, rather than regurgitating facets of what has previously been learnt, and implement it in the appropriate context [9]. The Berlin Questionnaire has not been adapted for use in disciplines other than medicine [10].

Both the Berlin Questionnaire and the Fresno test (along with its adaptions) are classified as the only level 1 instruments currently with the ability to assess competency in EBM [4]. Level 1 instruments are defined as those that have robust psychometric properties and have the ability to discriminate between different EBM levels, or expertise [4]. As level 1 instruments, the Fresno test and Berlin Questionnaire have the ability to discriminate between different levels of expertise across users, possess robust psychometric properties and examine skills and knowledge that users would require in undertaking a realistic task related to practising EBM [4,10].

Training in EBM has become commonplace across medical curricula worldwide [11]. Multiple choice questions (MCQs), extended matching questions (EMQs), critical appraisal topics, essays and objective structured clinical examinations (OSCEs) are all forms of assessment that may examine, to a certain degree, learner competency in EBM [12-14]. None of the above mentioned modes of assessment have been developed and validated to specifically assess EBM competency in medical trainees. Both the Berlin Questionnaire and Fresno test are limited in their ability to measure EBM competency within a medical curricula. Neither instrument has been validated in a sample of medical trainees. The Fresno test requires 60 minutes for users to complete, and a larger investment of time and resources for its grading. Conversely, the Berlin Questionnaire can generally be completed within 15–20 minutes, and poses a smaller burden on time regarding grading due to use of 15 MCQs. Significantly, neither of these level 1 instruments examines competency across all 5 EBM steps.

The purpose of this study was to develop and validate an instrument that evaluates medical trainees’ competency in EBM across all examinable steps of the EBM process. The fifth step of the EBM process is evaluation, which has been suggested is best assessed through observation [3]. The proposed ‘Assessing Competency in EBM’ (ACE) tool focuses on assessing competency in steps 1 to 4 of the EBM process.

## Methods

### ACE tool description

The ACE tool presents users with a short patient scenario from which a clinical question is derived. Users are then presented with a search strategy (designed to identify a randomised controlled trial) and a hypothetical article extract. Users are required to work through 15 questions (answering yes or no), with each question representing one of the four steps of EBM (formulation of the clinical question, search of the literature, critical appraisal and application of the evidence to the patient) (Additional file 1). Items 1–11 assess knowledge and skills relevant to EBM, whilst items 12–15 assess attitudes relevant to the implementation of EBM in clinical practice.

### ACE tool development

The ACE tool was developed by five experienced teachers in EBM (DI, RBN, PG, JT, EV). DI developed the initial version of the ACE, with the remaining four authors modifying the tool to ensure that all steps of the EBM process were adequately addressed. The 15 items assess four of the steps associated with EBM – the exception being the last step of evaluation. Items 1 and 2 relate to step 1 (asking the answerable question), items 3 and 4 relate to step 2 (searching the literature), items 5–11 relate to step 3 (critical appraisal) and items 12–15 relate to step 4 (applying the evidence to the patient scenario). A variety of the items were based on existing critical appraisal templates and modified accordingly for the development of the ACE tool [15,16]. Content and face validity of the ACE tool was established through an iterative process by consensus expert opinion [17].

A cross-sectional, convenience sample of 342 medical trainees from Monash University participated in the study. Three trainee cohorts were recruited to represent different levels of EBM competency. Trainees were divided into three cohorts according to the number of years training in EBM – (i) EBM-novice (less than two years training), (ii) EBM-intermediate (three years training), and (iii) EBM-advanced (four years training). The course content is consistent across all years of training with emphasis on the key steps in EBM [18]. Each year subsequently builds on previous year’s work through a spiral approach to learning [18]. Participants were given an opportunity to complete the ACE tool online, with a 60-minute time limit provided. Answers for the ACE tool were scored one for a correct answer and zero for an incorrect answer, for a maximum score of 15. This study was approved by the Monash University Human Research Ethics Committee (MUHREC).

### Data analysis

Construct validity was determined by analysis of variance for linear trends for mean scores across the three EBM groups. Post-hoc analysis was performed by assessing mean difference between EBM groups. Item difficulty was determined by comparing pass rates across the three EBM groups for individual items via chi-square analysis. Internal reliability was determined by item-total correlation (ITC) using Pearson product – moment correlation coefficients. An ITC ≥ 0.15 is considered acceptable as it examines the degree to which all test questions on the test measure a single construct [19]. Internal consistency was measured via Cronbach’s alpha [20]. A Cronbach’s alpha ranging from 0.6-0.7 is considered to demonstrate ‘acceptable’ internal consistency, 0.7-0.9 ‘good’ internal consistency and >0.90 as ‘excellent’ internal consistency [21,22].

Item discrimination index (IDI) examines the ability of each item to discriminate between participants overall high scores and those with overall low scores. IDI was calculated for each item by separating participant total scores into quartiles, then subtracting the proportion of participants in the bottom quartile who correctly answered the item from participants in the top quartile who correctly answered the item correctly [23]. IDI ranges from a negative (−1) to positive (1), with an IDI > 0.2 considered satisfactory [24]. Time to completion was analysed via analysis of variance. A p-value <0.05 was considered statistically significant for all statistical analysis. Data were analysed using STATA version 11. A box-and-whisker plot was utilised to depict the median and quartile values of the data. Outliers were plotted as individual plots.

## Results

A total of 342 participants, consisting of 98 EBM-novice, 108 EBM-intermediate and 136 EBM-advanced, enrolled in the study and completed the ACE tool. The overall properties of the ACE tool are summarised in Table 1.

**Table 1:** Summary of the properties of the ACE tool

**Test property**	**Measure used**	**Acceptable results**	**Performance of the ACE tool**
**Content validity**	Expert opinion	Test covers steps 1–4 of EBM	Acceptable
**Item difficulty**	Percentage of candidates who correctly answered the question	Wide range of results allows implementation across a wide range of participants including novice to expert	Ranged from 36% to 84%
**Internal consistency**	Cronbach’s alpha	Cronbach’s alpha 0.6-0.7 is considered acceptable, 0.70-0.90 good and >0.90 excellent	Cronbach’s α = 0.69
**Internal reliability**	Item-total correlation (ITC)	≥0.15 is considered acceptable	Ranged from 0.14 to 0.20 all items apart from three (0.03, 0.04 & 0.06)
**Item discrimination index**	Item discrimination index (ranges from −1.0 to 1.0)	All items should be positively indexed, ≥ 0.20 is considered acceptable	Ranged from 0.37 to 0.84
**Construct validity**	Mean scores three participant cohorts (EBM-novices, EBM-intermediate and EBM-advanced) compared by ANOVA	Significant differences in mean scores with EBM-advanced > EBM-intermediate > EBM-novice	On a 15-point test, mean scores were 8.6 EBM-novice; 9.5 EBM-intermediate; and 10.4 EBM-advanced (p < 0.0001)

### Construct validity

**Figure 1:**
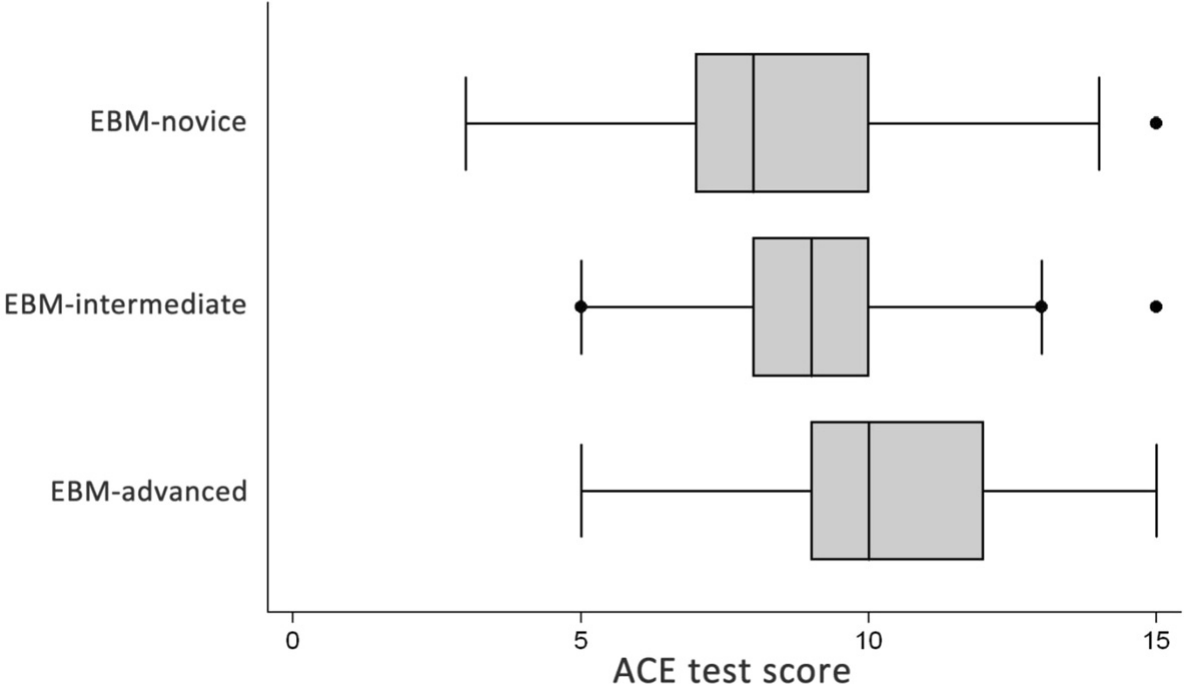
**Box and whisker plot of ACE scores across EBM-novice (n = 98), EBM-intermediate (n = 108) and EBM-advanced (n = 136) participants.** ANOVA for linear trends demonstrated statistical significance (p < 0.0001).

The three groups with different levels of training in EBM had three distinct scores on the ACE tool. Total mean (±SD) scores across the three groups were; EBM-novice: 8.6 ± 2.4, EBM-intermediate: 9.5 ± 1.8 and EBM-advanced: 10.4 ± 2.2. A statistically significant linear trend for sequentially improved mean score corresponding to the level of training was observed (p < 0.0001) (Figure 1). Mean differences (95% CI of difference) between groups were; EBM-novice vs EBM-intermediate was −0.85 (−1.55 to −0.01), EBM-novice vs EBM-advanced was −1.8 (−2.48 to −1.14), and EBM-intermediate vs EBM-advanced was −0.96 (−1.61 to −0.31).

### Individual item analysis

Individual item results for internal reliability, item difficulty, item discrimination and construct validity are described in Table 2. Relative difficulty (item difficulty) of each item was broad and ranged from a pass rate of 36% (item 15) to 84% (item 3). Internal reliability (ITC) was acceptable for the majority of items apart from three (item 5, item 6 and item 8). The ability of the ACE tool to discriminate between participants with high versus low overall scores (IDI) was excellent, with items ranging from 0.37 to 0.84. Statistically significant pass rates were observed between groups for all items apart from items 3 and 4. The Cronbach’s alpha coefficient for internal consistency was measured as 0.69, which was considered to demonstrate ‘acceptable’ internal consistency estimate of the reliability of the item scores.

**Table 2:** Individual item analysis was performed to assess item IDI, ITC and difficulty

Step*	**Item**	**IDI**	**ITC**	**Novice pass rate (%)**	**Intermediate pass rate (%)**	**Advanced pass rate (%)**	**Overall pass rate (%)**	**P-value**^**+**^
1	1	0.70	0.15	41	96	71	69	0.0001
1	2	0.74	0.15	58	94	70	74	0.0001
2	3	0.84	0.15	83	91	79	84	0.059
2	4	0.61	0.19	65	41	74	60	0.0001
3	5	0.70	0.06	57	88	65	70	0.0001
3	6	0.42	0.03	15	97	19	43	0.0001
3	7	0.49	0.15	52	33	61	49	0.0003
3	8	0.66	0.04	83	17	96	66	0.0001
3	9	0.81	0.14	75	72	95	81	0.0001
3	10	0.67	0.20	56	47	93	66	0.0001
3	11	0.59	0.14	38	88	52	59	0.0001
4	12	0.79	0.17	67	84	84	79	0.003
4	13	0.76	0.16	79	54	94	76	0.0001
4	14	0.40	0.16	62	7	51	40	0.0001
4	15	0.37	0.17	32	37	40	36	0.49

Chi-square analysis for difference in pass rates between EBM-novice, intermediate and advanced groups. IDI = item discrimination index, ITC = item-total correlation.

*Items 1 and 2 relate to EBM step 1 (asking the answerable question), items 3 and 4 relate to step 2 (searching the literature), items 5–11 relate to step 3 (critical appraisal) and items 12–15 relate to step 4 (applying the evidence to the patient scenario).

### Time to completion

Mean time (±SD) for all participants to complete the ACE tool was 12.8 (±8.6) minutes. Completion times across the specific groups were; EBM-novice 12.8 (±8.31) minutes, EBM-intermediate 13.6 (±9.2) minutes and EBM-advanced 12.1 (±8.3) minutes. No significant difference in time taken to complete the ACE tool between the groups was observed (p > 0.05).

## Discussion

This study demonstrates that the ACE tool has moderate validity and internal reliability as instrument in assessing EBM competency in medical trainees. The ACE tool provides users with an instrument that can measure competency across the first 4 steps of EBM. The ACE tool attempts to integrate aspects from both the Berlin Questionnaire [6] and the Fresno tool [5] in providing users with an assessment that measures deep learning, whilst integrating skills measuring a users’ ability to ask, acquire, appraise and apply evidence across a realistic patient scenario. We also believe that the ACE tool is the first to offer a dichotomous outcome measure, compared to previous iterations of assessment tools that have utilised a range of open-ended and multiple-choice question responses [4].

The CREATE framework classifies user ‘knowledge’ and ‘skills’ in EBM as constructs that may be assessed via validated assessment instruments. The other categories (reaction to educational experience, attitudes, self-efficacy, behaviours, and benefits to patients) are more appropriately assessed using a combination of self-report, opinion, active monitoring and patient-oriented outcomes [3]. The ACE tool satisfies the requirements, as per the CREATE framework, for examining user knowledge and skills across the steps 1–4 of the EBM process. Step 5, ‘evaluation’, relies in part on users reflecting on the success of the EBM process in the patient scenario, and what aspect (if any) should be altered for future use. Assessing reflection as an outcome with a dichotomous response (as per the ACE tool) or any other multiple choice version is not appropriate and should be best measured through other open-ended outcome assessment.

A key strength of the ACE tool is the ability to assess users’ deep-learning ability. The Berlin Questionnaire assesses user competency predominantly in critical appraisal, with a large emphasis on calculating and interpreting biostatistics. Limited emphasis is placed on assessing other key aspects of the EBM process including constructing an answerable question, acquiring and applying the evidence. The ACE tool is comprehensive in the 4 EBM steps that it assesses, yet is simple to implement. Unlike the Fresno test, the ACE tool can be completed quickly by users (less than 15 minutes on average to complete) and the dichotomous response structure doesn’t require interpretation from raters.

Several limitations of the ACE tool must be considered. Three of the items (5, 6 and 8) were identified as poor performers regarding internal reliability. These three items correspond to questions about critical appraisal, with specific reference to selection and performance bias. Future refinement of the ACE tool could consider re-phrasing item 5 to further differentiate the question as one that is concerned with investigating the baseline characteristics/distribution between groups (i.e. selection bias). Items 6 and 8 relate to selection bias (allocation concealment) and performance bias. In its current form, the ACE tool, with only one patient scenario, search strategy and article, does not necessarily permit repeated use on the same cohort. Future versions of the ACE tool will require alternate scenarios to account for the potential impact of recall bias during testing.

Whilst an overall consistent linear performance across EBM competency (i.e. novice < intermediate < advanced) was observed, a similar trend was not evident across the 15 items. A total of 10 items returned unusual presentations, with both the EBM-novice group demonstrating a larger effect than the EBM-intermediate group, and similarly the EBM-intermediate group demonstrating a larger effect than the EBM-advanced group. Some of this variation may be attributed to the differences in emphasis placed on EBM concepts as perceived by medical students at these different competency levels. In the current curriculum, a strong emphasis is placed on research methodology and biostatistics in the formative years. A greater emphasis on constructing a question, searching the literature and critical appraisal is placed in the intermediate years, whilst the final years concentrate on application of EBM principles in clinical practice. This variation on ‘spot-lighting’ certain aspects of the EBM continuum may have contributed to the non-linear trends observed within the 10 items.

The current version of the ACE tool has been developed and validated to answer questions relating to ‘therapy’. In practice clinicians will encounter a variety of patient scenarios exclusive to therapy including aetiology, harm, diagnosis and prognosis. Further iterations of the ACE tool are required to incorporate patient scenarios that examine competency across those clinical questions. The inclusion of these scenarios would provide a complete suite of instruments to assess EBM competency, regardless of patient scenario.

## Conclusion

The 15-item ACE tool is a valid and reliable instrument for assessing the EBM competency of medical trainees. Implementation of the ACE tool is simple and provides educators with a reliable evaluation of trainees’ competency across key constructs in EBM. Further development of the ACE tool across different clinical disciplines will provide valuable information on the validity of the ACE tool in users other than medical trainees.

## Competing interests

DI coordinates the EBM program for the MBBS degree at Monash University.

## Authors’ contributions

DI contributed to the study concept and design, data collection and analysis and interpretation of the data. RBN, PG, JT and EV contributed to the study concept and design, and the interpretation of the data. All authors contributed to the drafting and revision of the article and approved the final manuscript for publication.

## Authors’ information

DI is Associate Professor at the School of Public Health & Preventive Medicine, Monash University.

RBN is Professor at the Jeffrey Cheah School of Medicine and Health Sciences, Monash University, Malaysia.

PG is Professor at the Faculty of Health Sciences and Medicine, Bond University, Australia.

JKT is Associate Professor at the Division of Biokinesiology and Physical Therapy, University of Southern California, USA.

EV is Associate Professor at the Gippsland Medical School, Monash University, Australia.

## Pre-publication history

The pre-publication history for this paper can be accessed here:

http://www.biomedcentral.com/1472-6920/14/114/prepub

## Supplementary Material

Additional file 1 Assessing Competency in Evidence Based Medicine (ACE) tool. Click here for file
