# Supplementary material for: Development and validation of the ACE tool: assessing medical trainees’ competency in evidence based medicine
Source: BMC Med Educ. 2014 Jun 9;14:114. doi: 10.1186/1472-6920-14-114 (PMC4062508; doi:10.1186/1472-6920-14-114)
Supplement: Additional file 1 — Assessing Competency in Evidence Based Medicine (ACE) tool. [file 1472-6920-14-114-S1.pdf]

Assessing Competency in Evidence Based Medicine (ACE tool)

Read through the following information on patient scenario, clinical question, search strategy and article extract before answering the following set of questions.

**Asking an answerable question**	**Yes**	**No**
1. Are all PICO elements described in the patient scenario?		
2. Does the question constructed post-scenario provide a focused, foreground question?		
**Searching the literature**	**Yes**	**No**
3. Will the search strategy (to be used in Medline) retrieve relevant studies relating to the question?		
4. Does the search strategy utilise appropriate MeSH/keywords and Boolean operators correctly and effectively?		
**Appraising the evidence**	**Yes**	**No**
5. Was there sufficient information to determine the representativeness of the study participants?		
6. Was the method of participant allocation to intervention/exposure and comparison adequate?		
7. Was any form of adjustment required?		
8. Were all participants blinded to the treatment/exposure?		
9. Were all investigators blinded to the treatment/exposure?		
10. Were all outcome assessors blinded to the treatment/exposure?		
11. Were all patients analysed in the groups to which they were randomised?		
**Applying the evidence**	**Yes**	**No**
12. Does the patient in the scenario share similar characteristics/circumstances to participants in the study?		
13. Is the treatment/therapy feasible in the clinical setting of the scenario?		
14. Were all clinically important outcomes considered?		
15. Do the likely benefits of the treatment/therapy outweigh any potential harms and costs?		

Patient scenario

"Jane is a 42 year-old female Caucasian, who lives with her partner in metropolitan Melbourne, Australia. Jane is a lawyer, who quit smoking three years ago, after being a 'pack-a-day' smoker since her early 20s. Since her late 30s, Jane has received treatment for hypertension. Her medical history is otherwise unremarkable. At her most recent visit to her family doctor, Jane mentions that she has seen reports on the television about a new study investigating the preventive effects of aspirin. She has heard that aspirin may be beneficial in protecting against cardiovascular disease. Jane wonders whether she should be taking aspirin, given her history with hypertension, but wonders whether also being a diabetic might negate any benefit."

Clinical Question

"Is aspirin effective in reducing the risk of cardiovascular disease?"

Search strategy

Item	Search	Results
1	Aspirin.mp	52620
2	exp Aspirin/	38658
3	1 OR 2	52620
4	exp Cardiovascular Diseases/	1874575
5	cardiovascular.mp.	352938
6	4 OR 5	2003546
7	Hypertension/ or hypertension.mp.	355606
8	diabetes.mp. or Diabetes Mellitus/	393986
9	3 AND 6 AND 7 AND 8	905
10	Limit to randomised controlled trials	75

Article extract (hypothetical article)

A randomised controlled trial of aspirin for the prevention of cardiovascular disease

Background

Aspirin is effective in the treatment of acute myocardial infarction and prevention of cardiovascular disease in men and women. Previous studies on the use of aspirin in primary prevention of cardiovascular disease have demonstrated a positive effect in men, yet the benefit in women remains uncertain. The aim of this study was to assess the effect of aspirin in the prevention of cardiovascular disease in women.

Methods

The study design was a randomised, double-blinded, placebo-controlled, trial of low-dose aspirin in the prevention of cardiovascular disease in women. The design of the study has previously been described in detail. In brief, between January 2002 and January 2012, letters of invitation were mailed to 500,000 women in the greater city of Melbourne, Victoria, Australia. A total of 63,250 volunteered to enrol in the study. Women were eligible if they were 40 years of age or older; had no history of coronary heart disease, cerebrovascular disease, no previous side-effects to taking aspirin and were not currently taking aspirin or any non-steroidal anti-inflammatory drug (NSAID) medication. A total of 31,150 women met the inclusion criteria of which 15,100 were randomised (through the generation of a computer generated scheme) to receive aspirin and 15,102 were randomised to receive the placebo. Written informed consent was obtained from all participants prior to commencement in the study. The trial was approved by the ethics board at the governing hospital and university institution. Participants in both groups were required to present every 6 months at the study site centre for assessment and to receive their medication. Medication was provided by the site pharmacy, which allocated identical appearing aspirin and placebo tablet in blister packs to the study's participants independent to the study's investigators. All participants were followed for myocardial infarction, stroke or death from cardiovascular causes. Medical records were obtained for all women in whom a cardiovascular event was recorded. These records were reviewed by an end-point committee, consisting of study investigators blinded to the treatment. The primary end point was cardiovascular events – a combination of myocardial infarction, stroke or death from cardiovascular causes. Only confirmed end-points of cardiovascular events were included in this study. Cox proportional hazard models were used to calculate hazard ratios and 95% confidence intervals for the comparison of event rates in the aspirin and placebo groups after adjustment for age.

Results

Both aspirin and placebo groups were similar with respect to baseline characteristics (Table 1). The average duration of follow-up from randomisation to the end of the trial was 4.2 years (range, 2.3 to 5.0 years). Throughout the duration of the trial, drop-outs occurred. Data presented is based on participants that completed the trial during the study period. A total of 422 women in the aspirin group and 478 women in the placebo group had a cardiovascular event (Hazard Ratio, 0.83; 95% confidence interval, 0.77 to 1.01). There was no evidence that any of the cardiovascular risk factors considered, except smoking status and hyperlipidemia, modified the effect of aspirin on the primary end-point.

Discussion

In this large study, involving 63,250 women, a 100 mg daily dose of prophylactic aspirin is associated with a reduced risk of major cardiovascular events. No significant evidence was found that age, hypertension, diabetes or BMI modified the effect of aspirin. Middle aged women who adhere to a daily low dose of aspirin can significantly reduce the risk of cardiovascular disease. The rate of benefit is large, with a cardiovascular event prevented for every 269 women treated with aspirin.

Table 1. Baseline characteristics			
	**Aspirin (N=15,100)**	**Placebo (N=15,102)**	**Total (N=30,202)**
**Age (years)**			
**(mean±SD)**	55.3±8.0	54.9±8.0	55.1±8.0
**40-50 (%)**	50.2	50.1	50.1
**51-60 (%)**	42.9	43.0	43.0
**>61 (%)**	6.9	6.9	6.9
**Smoking status**			
**Current (%)** [+]	15.0	14.7	14.9
**Past/never (%)**	85.0	85.3	85.1
**Body mass index (kgm^{-2})**			
**(mean±SD)**	25.1±4.3	25.3±4.3	25.2±4.3
**≤25.0 (%)**	48.8	48.8	48.8
**25.1-29.9 (%)**	32.1	32.2	32.2
**≥30.0 (%)**	19.1	19.0	19.0
**Hypertension**			
**Yes (%)**	25.0	24.9	25.0
**No (%)**	75.0	75.1	75.0
**Diabetes**			
**Yes (%)**	2.3%	2.2%	2.2%
**No (%)**	97.7%	97.8%	97.8%
**Hyperlipidemia**			
**Yes (%)**	27.3	27.2	27.2
**No (%)**	72.7	72.8	72.8

Mean differences tested using independent t-test; proportional differences tested using the chi square test. [+]significantly different at $p<0.05$.

Table 2. Hazard ratios of cardiovascular events, related to baseline characteristics				
	**Total number**	**Aspirin**	**Placebo**	**HR (95% CI)**
**Age (years)**				
**40-50**	15131	122	142	0.86 (0.67-1.09)
**51-60**	12987	148	166	0.89 (0.71-1.13)
**>61**	2084	152	170	0.90 (0.74-1.11)
**Smoking status**				
**Current**	4500	159	140	1.12 (1.00-1.40)
**Past/never**	25702	263	338	0.78 (0.66-0.92)
**Body mass index (kgm^{-2})**				
**≤25.0**	14738	181	208	0.87 (0.71-1.06)
**25.1-29.9**	9725	150	169	0.97 (0.71-1.11)
**≥30.0**	5739	91	101	0.90 (0.68-1.20)
**Hypertension**				
**Yes**	5051	221	250	0.89 (0.75-1.06)
**No**	25151	201	228	0.87 (0.73-1.06)
**Diabetes**				
**Yes**	664	58	62	0.94 (0.68-1.31)
**No**	29538	364	416	0.87 (0.76-1.01)
**Hyperlipidemia**				
**Yes**	8214	196	168	1.15 (1.04-1.48)
**No**	21988	226	310	0.73 (0.62-0.87)

Assessing Competency in Evidence Based Medicine (ACE tool)
Answers

Asking an answerable question	Yes	No
1. Are all PICO elements described in the patient scenario?	✓	
2. Does the question constructed post-scenario provide a focused, foreground question?		✓
**Searching the literature**	**Yes**	**No**
3. Will the search strategy (to be used in Medline) retrieve relevant studies relating to the question?	✓	
4. Does the search strategy utilise appropriate MeSH/keywords and Boolean operators correctly and effectively?	✓	
**Appraising the evidence**	**Yes**	**No**
5. Was there sufficient information to determine the representativeness of the study participants?	✓	
6. Was the method of participant allocation to intervention/exposure and comparison adequate?		✓
7. Was any form of adjustment required?		✓
8. Were all participants blinded to the treatment/exposure?	✓	
9. Were all investigators blinded to the treatment/exposure?	✓	
10. Were all outcome assessors blinded to the treatment/exposure?	✓	
11. Were all patients analysed in the groups to which they were randomised?		✓
**Applying the evidence**	**Yes**	**No**
12. Does the patient in the scenario share similar characteristics/circumstances to participants in the study?	✓	
13. Is the treatment/therapy feasible in the clinical setting of the scenario?	✓	
14. Were all clinically important outcomes were considered?		✓
15. Do the likely benefits of the treatment/therapy outweigh any potential harms and costs?		✓

Reasoning for answers

1. All PICO elements are provided in the patient scenario.
2. PICO question is not focused ("Is aspirin effective in reducing the risk of cardiovascular disease?"), no mention of patient aspects only intervention and outcome.
3. Search strategy will retrieve relevant studies.
4. Appropriate use of MeSH/keywords and Boolean operators.
5. Information regarding source, eligible and participant population was made available.
6. Randomisation is mentioned, but no detail on how this randomisation process was achieved or if allocation concealment was achieved.
7. No adjustment necessary as baseline characteristics are similar.
8. Participants were blinded to treatment (use of pharmacy to dispense identical looking pills).
9. Investigators blinded through use of third party (pharmacy) to dispense intervention and placebo tablets.
10. Outcomes assessed by end-point committee, consisting of study investigators blinded to the treatment.
11. Drop-outs occurred, no ITT analysis performed, analysis only on participants completing the trial (per protocol analysis).
12. Age, smoking status, hypertension and diabetes all relevant to the patient, and reported in the study.
13. Treatment (aspirin) is feasible in the clinical setting (i.e. general practice, metropolitan Melbourne).
14. Clinically important outcomes such as side effects of aspirin were not reported.
15. Hazard ratio does not report a statistically significant decrease in the risk of cardiovascular events. No evidence on potential harms, or costs.
